# Culture and Use of Mesenchymal Stromal Cells in Phase I and II Clinical Trials

**DOI:** 10.4061/2010/503593

**Published:** 2010-10-31

**Authors:** Bourin Philippe, Sensebé Luc, Planat-Bénard Valérie, Roncalli Jérôme, Bura-Rivière Alessandra, Casteilla Louis

**Affiliations:** ^1^EFS-PM, Laboratoire d'Ingénierie Cellulaire, GECSoM, 75 rue de Lisieux, 31300 Toulouse, France; ^2^Service Recherche, EFS-CA, GECSoM, 2 boulevard Tonnellé BP52009, 37020 Tours Cedex 1, France; ^3^UMR 5241 Métabolisme, Plasticité et Mitochondrie, BP84225, 31432 Toulouse Cedex 4, France; ^4^Service de Cardiologie, CHU Rangueil, TSA 50032 1 avenue Jean Poulhes, 31059 Toulouse Cedex 9, France; ^5^Service de Médecine Vasculaire, CHU Rangueil, TSA 50032 1 avenue Jean Poulhes, 31059 Toulouse Cedex 9, France

## Abstract

Present in numerous tissues, mesenchymal stem cells/multipotent stromal cells (MSCs) can differentiate into different cell types from a mesoderm origin. Their potential has been extended to pluripotency, by their possibility of differentiating into tissues and cells of nonmesodermic origin. Through the release of cytokines, growth factors and biologically active molecules, MSCs exert important paracrine effects during tissue repair and inflammation. Moreover, MSCs have immunosuppressive properties related to non-HLA restricted immunosuppressive capacities. All these features lead to an increasing range of possible applications of MSCs, from treating immunological diseases to tissue and organ repair, that should be tested in phase I and II clinical trials. The most widely used MSCs are cultured from bone marrow or adipose tissue. For clinical trial implementation, BM MSCs and ADSCs should be produced according to Good Manufacturing Practices. Safety remains the major concern and must be ensured during culture and validated with relevant controls. We describe some applications of MSCs in clinical trials.

## 1. Introduction

From the end of the 1960s to the beginning of the 1970s, a Soviet scientist, Alexander Friedenstein, discovered a population of adherent cells in bone marrow (BM) that could differentiate into osteoblasts, chondrocytes, and hematopoietic stromal supportive cells [1]. The cells had a fibroblast shape, and when seeded at low density, some could form clonal colonies, which suggested the presence of precursor cells, the colony-forming unit-fibroblasts. Because the cells were capable of differentiating into various lineages of the mesoderm, they were named mesenchymal stem cells (MSCs) [2]. The stemness status and particularly the long-term self-renewal potential of MSCs has not been definitely established, so the preferred term is multipotent mesenchymal stromal cells [3], both terms being abbreviated to MSCs. In general, MSCs refer to native stem cells present *in vivo* in BM and to derived cultured cells.

Cultured MSCs are a mix of cells ranging from progenitors to mature stromal cells. Besides their differentiation potential, MSCs have an immunosuppressive effect both *in vitro* and *in vivo* by acting on all immune effectors [4]. However, the role of MSCs in tissue repair is not restricted to their differentiation potential or immunosuppressive effects. Indeed, MSCs have consistent trophic effects mediated by the wide range of growth factors and cytokines they produce [5]. 

These biological properties of MSCs rapidly led to investigation of their use in cell-based therapy by the middle of the 1990s. Caplan's team was the first to intravenously inject autologous, cultured MSCs in patients during a safety assessment trial [6]; up to 50 × 10^6^ MSCs could be safely injected in humans. Later, injected MSCs were used in clinical trials to treat diseases such as osteogenesis imperfecta [7], metachromatic leukodystrophy [8], acute myocardial infarction [9], and graft-versus-host disease (GVHD) [10]. MSCs were also implanted to treat bone defects [11, 12]. More than 50 clinical trials related to MSCs have been reported at http://www.clinicaltrials.gov.

MSC populations with similar properties are found in almost all tissues in mammals and humans [13, 14]. Among them, adipose tissue is the most promising source of MSC-like cells suitable for clinical trials. Indeed, since the description of adipose-derived stromal cells (ADSCs) by Zuk and colleagues, in 2001, the large amount of data generated has shown adipose tissue to be the richest source of mesenchymal progenitor cells (ADSCs are at least 100 times more abundant in adipose tissue than are MSCs in BM). ADSCs and MSCs share many characteristics [15] but also differences in protein and function [16] (Table 1). For example, ADSCs have greater angiogenic potential than do MSCs [17]. ADSCs were used in clinical trials as soon as 5 years after their description [18, 19] and more than 10 clinical trials have been reported at http://www.clinicaltrials.gov. The clinical use of MSCs from other sources, especially the fetus, is far less advanced. 

In this paper, we report on the current experience in the use of MSCs and ADSCs. First we discuss culture requirements and safety concerns, and then describe several ongoing clinical trials.

## 2. Culture Requirements: From Bench to Bedside

### 2.1. Culture Medium

The proof of concept and original data obtained *in vitro* and *in vivo* in the field of cell therapy are generally acquired with cells derived from culture protocols established for research use and do not necessarily follow Good Manufacturing Practices (GMP) rules. Thus, before injecting cells in humans, the first step is to replace research reagents with products suitable for human use. At this step, the two main problems are to accurately adapt the culture protocol with appropriate reagents and to prove that the resulting human cell culture exhibits all the properties described in the proof of concept. 

No well-established rule exists to adapt the culture protocol, but the adaptation must be progressive (change one parameter at a time), with a careful validation of the therapeutic properties of the cells at each step. Because animal experiments must be sparingly used (referred to the 3R statement of the European Community: reduce replace refine), *in vitro* validation tests must be preferred and developed in order to limit animal experimentation. For example, angiogenic properties can be estimated by the dosage of angiogenic factors released in the culture medium and/or by measurement of *in vitro* vessel-like structure formation. Whatever the tests, they must be closely related to the expected *in vivo* effects of the cells. At the end of the adaptation phase, the cell product must be tested *in vivo* in small-animal models and, if possible, large-animal models. We discuss later the relevance and validity of the animal model. 

Special attention should be paid to the risk of infectious disease transmission to humans by components in the culture medium. Consideration of this risk must also include the new variant of Creutzfeld-Jakob disease. Moreover, by using a culture medium containing proteins from an animal origin, MSCs may retain in their cytoplasm a substantial amount of xenogenic proteins. A standard preparation can represent 7 to 30 mg of fetal calf serum (FCS) proteins per 100 × 10^6^ MSCs [20]. This level of proteins may elicit immunologic responses *in vivo* that could explain some of the failures in MSC cell therapy [7, 20]. Such data led to an effort to decrease or eliminate the use of xenogeneic components in the culture media for clinical MSC preparations. Replacing FCS with human autologous or AB serum has been proposed, but both appear to be less effective than FCS. However, supplementing the medium with human AB serum and fibroblast growth factor 2 can overcome this deficit [21]. Alternatively, FCS can be replaced with human plasma enriched with growth factors contained in platelets, such as platelet-derived growth factor, endothelial growth factor, and vascular endothelial growth factor. These cytokines are strongly mitogenic for MSCs and have been used since the 1980s [22, 23]. They can be obtained by activation of platelets with thrombin or simply by a cycle of freezing/thawing of the plasma that disrupts the platelets and releases growth factors [24, 25]. In our experience, this substitute for FCS is effective for MSC and ADSC expansion *in vitro*: it reduces the cell doubling time by at least 30% and the cells retain their morphology and functions. 

The need to cultivate MSCs in a defined medium is important for homogeneity between cell production processes. In the 1990s, at least two teams described formulations allowing for cultivation of MSCs in a defined medium [26, 27]. Since then, some companies have developed similar defined media for MSC culture that unfortunately do not contain molecules inducing MSC adhesion. Thus, the culture process requires treatment to adsorb an attachment protein (fibronectin, collagen) on the culture surface. In addition, the formulation often is not disclosed, which prevents its use in clinical trials. Furthermore, these formulations do not include growth factors, which must be added to the medium, with the concern that they have not been produced with GMP. The following describes the culture of MSCs.

### 2.2. Culture Device: Closed Systems, a First Approach

**Figure 1:**
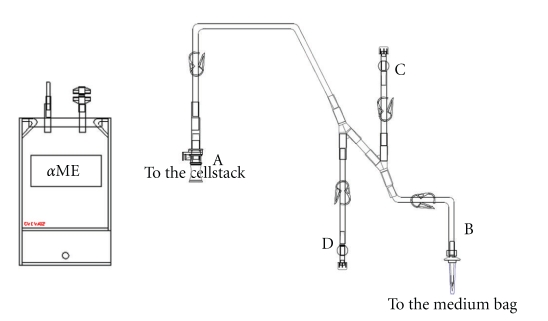
Set of tubing plus medium bag for cell seeding operation.

**Figure 2:**
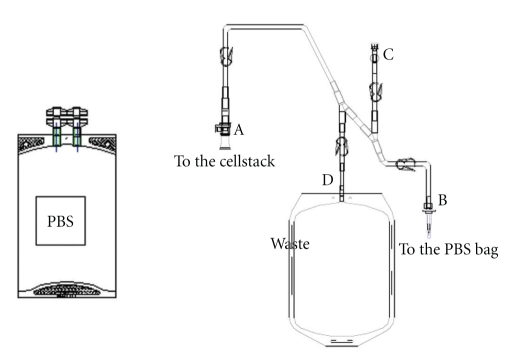
Set of tubing plus phosphate-buffered saline and waste bag for medium exchange.

**Figure 3:**
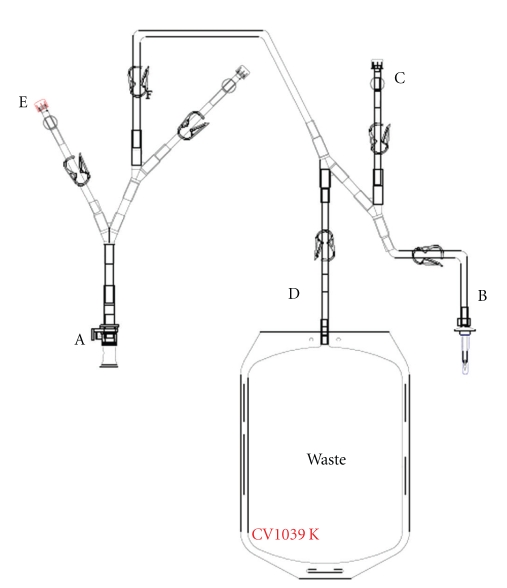
Set of tubing with waste bag for cell detachment.

The production of MSCs, which are adherent with contact inhibition requires substantial culture surfaces. As soon as the clinical trial requires more than 10^8^ MSCs, surface areas of culture exceeding 2000 cm^2^are needed, which corresponds to at least seven 300-cm² culture flasks; these are time consuming to handle and imply a nonnegligible risk of contamination. When a large number of cells are required, for example, for treating GVHD ([3 to 8] × 10^8^ MSCs per patient), the flask solution will become unmanageable because the required surface area exceeds 6000 cm². With some culture containers, reaching this surface area with a few units is impossible. Possible solutions are the CellStacks (Corning, the USA) and CellFactory (Nunc, Denmark) systems, which start from a unit surface area of 635 cm^2^ and offer the possibility of 2, 5, 10, and 40 stages per container. These devices can also be connected by tubes for performing various operations (e.g., culture initiation, medium exchange, cell harvesting) in a simple and protected way. In this case, all fluids must be contained in a form that ensures simple handling (sterile bags with suitable connections). We have partnered with a pharmaceutical company (MacoPharma, France) to develop a simple connection system in which the basic medium is prepared in sterile bags (Figures 1, 2, and 3). This system, adapted for 2- or 5-stage containers, ensures easy and rapid manipulation. Ten-stage containers and the associated fluid bags are so huge that they are cumbersome for a single technician to manage. All these systems that require connection steps cannot be strictly assimilated into a real closed system: they must always be handled in a very clean environment (class B zone) under a class A flow hood. A bioreactor that automates all the steps of the culture and allows for a real closed system can overcome these limitations. Such a machine must be versatile and allow for the elimination of unwanted nonadherent cells, as well as medium exchange and cell removal.

### 2.3. Safety Requirements: The Risk of Transformation

Regarding safety, the risk of transformation of MSCs during the culture process remains a major concern. Since the first report of spontaneous transformation of adipose-derived human MSCs, in 2005 [28], another team reported the same events with human BM MSCs [29]. In the first study, the transformation process required a long culture time and seemed to involve a mesenchymal-epithelial transition [30]. Concerning the second study of human BM MSC transformation, surprisingly, the process seemed shorter, with high frequency of transformation. However, for the first report, all the data concerning the transformation were later found to be related to contamination by an epithelial cancer cell line during the experimental procedures [31]. A similar retraction is under publication for the second report [32]. During the same time, using different techniques of controls, karyotype, and comparative genomic hybridization, numerous teams reported on genetically stable MSCs during culture [33, 34]. Moreover, in France, we used two different clinical-grade culture protocols, with aneuploidy features in a few productions, in two different clinical trials to study the significance of these features. We used karyotype and fluorescent *in situ* hybridization but also looked deeper at the molecular mechanisms involved in senescence and transformation. Clinical-grade cultured human BM MSCs, with or without aneuploidy, did not have any selective advantage, did not transform in culture and reached senescence with the normal evolution of adult cells [35]. In addition, tumors did not develop in immunocompromised mice injected with these MSCs. Finally, we demonstrated that the BM MSCs were not prone to genetic instability and did not easily transform during the normal culture process. These data and the retractions described previously strongly suggest that MSCs can be produced safely. However, these studies led to an improvement in controls for a focus on more accurate, relevant, and sensitive targets—investigating the expression and epigenetic status of the main genes of senescence and transformation pathways such as p53, p21, p16^ink4a^, hTERT, and c-myc.

### 2.4. Animal Models

As described previously, animal models are necessary for demonstrating safety and efficacy. In addition to causing potential lack of efficacy that could be encountered with chemical drugs (e.g., absence of the target, differences in metabolism, pharmacokinetics), human cell-based products elicit an immune reactive response in the host that rejects these cells. Thus, the cells must be tested in immunodeficient animals, which means rodents and most often mice. Nude or severe combined immunodeficiency (SCID) mice have been extensively used for this purpose, but they bear residual immune cells that could interfere with the human cells and thus bias the results. Thus, NOG mice or other very immunocompromised mice must be used, although the cost and difficulty of the experiments increase. Immunocompromised mice represent an aberrant immune context for many applications of cell-based therapy. Furthermore, the mouse may not be a relevant pathological model with their reduced life span as compared with humans and their size, which leads to difficult functional evaluation. For example, the heart rate in mice is about 300/s but only 80/s in humans; thus, intramyocardial injections may be problematic. Another solution could be to use a large-animal model and inject animal cells produced according to the same conditions as for human cells. Again, this solution is not completely satisfactory because of the real “active compound” (i.e., human cells are not tested, which implies a specific-cell production protocol because animal cells cannot be produced in the same rooms where human cells are produced). Despite these limitations, regulatory agencies require such validations. Thus, the validation experiments must be a compromise agreed upon by these authorities and must be different and specific to the treated condition.

## 3. Clinical Trials with MSCs and ADSCs

### 3.1. Acute Graft-Versus-Host Disease (aGVHD)

Because of the great immunomodulatory effects of MSCs *in vivo* and *in vitro* and since the first report by Le Blanc et al. [36], MSCs have been mainly used for treating or preventing aGVHD during allogeneic hematopoietic stem cell (HSC) transplantation. The efficiency of such therapy is greatest in liver and gut GVHD and in children [10, 37]. Some studies have suggested that cotransplantation of MSCs with HSCs can reduce the incidence of aGVHD [38]. In 2007, the Société Française de Greffe de Moelle et Thérapie Cellulaire (SFGM-TC) started a phase II, randomized placebo-controlled trial of corticosteroid-resistant aGVHD in patients receiving allogeneic HSC transplantation with or without coinjection of BM MSCs. MSCs were cultured according to a process developed by the SFGM-TC and validated by the French regulatory authority (AFSSaPS). The trial planned to enroll 78 patients. At month 5 and after two cell productions, karyotype analysis revealed clones with aneuploidy features not related to transformation [35], which led to suspension of the trial. Before the trial suspension, 11 patients were enrolled. This low number of patients did not allow for analysis of efficacy, but no enrolled patients receiving the cultured MSCs showed deleterious effects, even the one who received MSCs with aneuploidy. Potential late adverse events, including tumors, were never demonstrated. These observations are consistent with the lack of adverse side effects (transformation of MSCs, allosensitization by mismatched MSCs, increased incidence of infections) reported elsewhere during or immediately after infusion of MSCs [10, 38].

Further studies addressing use of MSCs with a randomized, large cohort of patients are under way [39]. Of note, treating aGVHD with MSCs for an immunosuppressive effect could lead to an increased frequency of relapse [40]. MSCs could be an efficient and safe way to treat and prevent aGVHD, but new studies should be implemented to focus on patients at risk for grade 2 to 4 aGVHD and for patients with visceral organ involvement.

### 3.2. Heart Failure

Cardiac failure after myocardial infarction represents poor prognosis and is associated with increased morbidity and mortality. Treatments for cardiac insufficiency have evolved well in recent years, but their purpose is to improve symptoms and prevent aggravation of the disease. Without possibility of revascularization of the myocardium, other therapies must be considered, such as injection of MSCs. The hope is to regenerate the cardiac muscle or to limit the ventricular remodeling by alternative mechanisms that call on the paracrine release of growth factors. Both small- and large-animal models have demonstrated the usefulness of MSC injection after acute and chronic myocardial infarction [41, 42]. Although many clinical trials have used uncultured BM cells, results of at least two clinical trials involving MSCs have been reported. In the first, a Chinese team harvested BM 8 days after the myocardial infarction, cultured it for 10 days, then reinjected the cells intracoronarily [43]. No side effects were observed, and the cardiac wall velocity and left ventricle ejection fraction values (LVEF) were improved in the treated group as compared with a control group. In the second randomized, double-blind, placebo-controlled clinical trial of 60 patients, 39 received cryopreserved allogeneic MSCs injected intravenously 1 to 10 days after myocardial infarction [44], with no adverse events or ectopic tissue formation observed. The group receiving MSCs showed significantly less arrhythmia. The LVEF was significantly improved in the subgroup with anterior myocardial infarction.

Because the risk of cardiac failure is high after myocardial infarction, we have begun a phase I clinical trial to evaluate the feasibility and safety of intramyocardial injection of BM MSCs in patients with ischemic cardiopathy. We include patients with myocardial infarction and stable symptomatic cardiac insufficiency (LVEF < 45%) of ischemic origin, without possibility of revascularization. From these patients, we harvest 15 ml BM, from which MSCs are selected by adherence to plastic and are amplified in culture during 17 days. The culture is done in devices that allow all steps to be performed in a near-closed system (see following discussion). The MSCs are injected (6 × 10^7^ MSCs) in the border zone of the infarction location. The main outcome of the trial is feasibility and safety of MSC intramyocardial injection at 1 month. The secondary outcomes are clinical, biological, and morphological effectiveness during 2-year follow-up. We aim to validate, for the first time, the feasibility and safety of intramyocardial injection of MSCs to improve the contractile function of the left ventricle and decrease morbidity of patients with cardiac insufficiency. Two patients have received the cells and have shown no adverse events.

### 3.3. Limb Ischemia

Critical limb ischemia (CLI) is a peripheral arterial disease with different grades described by Fontaine. Grades III and IV are characterized by chronic, ischemic pain at rest and ischemic skin lesions, either ulcers or gangrene. This clinical diagnosis can be confirmed by hemodynamic parameters such as ankle or toe systolic pressure. The frequency is around 32.5‰  before 40 years of age and 71‰  above 50 years. The estimated annual incidence of CLI ranges from 500 new cases per million in the European Union to 1 000 per million in the United States, with diabetes being the greatest risk factor. About 60% of patients should undergo revascularization whenever technically possible to prevent limb loss. The amputation rate for patients is about 20%. Because of the inefficiency of therapeutics when revascularisation is not possible, additional limb-saving strategies, including cell therapy, are required [45].

Results of a single clinical trial of CLI involving autologous BM mononuclear cells have been published. In total, 46 patients received intramuscular injections of (0.9 to 2.8) × 10^9^ BM mononuclear cells retrieved from patients under general anesthesia. The results were encouraging: 70% of patients showed improved oxygen tension, a significant decrease in pain and increased mobility [46]. Half of the patients reported no pain. The first positive effects appeared as soon as week 4, with stabilization at 6 months after treatment. Follow-up revealed significant improvement in leg pain, ulcer size, and pain-free walking distance maintained during at least 2 years after the therapy, although the ankle brachial index and transcutaneous oxygen pressure value did not change significantly [47].

From our preclinical data obtained from animal models, we began a phase I monocentric clinical trial to assess the feasibility and safety of intramuscular injections of autologous ADSCs for patients with CLI of the leg with no possibility of revascularization. We aimed to investigate the possibility of preventing amputation and more largely, decreasing morbidity. With patients under local anaesthesia, 30 g of adipose tissue is sampled by liposuction. After digestion and centrifugation of samples to separate mature, floating adipocytes from stromal cells, ADSCs are selected by adherence to plastic and are amplified in culture in a near-closed system for 2 weeks. The ADSC are injected (10^8^ cells) intramuscularly at 45 points in all the muscles of the leg by use of a grid.

The main outcome is feasibility and safety as assessed by observations of local necrosis, thrombosis, local and general infection or inflammation up to month 6 after ADSC injections. The secondary outcomes are wound healing, lack of amputation, oxygen tension at the toe or ankle, and pain. The trial aims to validate, for the first time, the feasibility and safety of the intramuscular injection of ADSCs to improve vascularity in the leg. Three patients have received ADSC injections and have shown no serious adverse events. This trial needs to include 6 more patients.

## 4. Future Directions

### 4.1. Allogeny

MSCs are well known to have an inhibitory effect on immune cell proliferation *in vitro *and* in vivo* [48, 49]. This observation has opened new perspectives concerning the use of MSCs in the context of allogenicity, for creating cell banks and for treating disease when there is no time to purify and expand cells. The results obtained with allogeneic cells are similar to those obtained with autologous cells. For the heart, intramyocardial injections and systemic delivery of allogeneic MSCs have been found to preserve myocardial viability and improve local and global heart function in rodents and pigs [50–52]. This finding is associated with decreased scar size and fibrosis and increased angiogenesis [53]. Most of the effects seem to be due to paracrine activity [44] and modulation of proinflammatory, proangiogenic, and immunomodulatory molecules by the peri-infarcted myocardium [54, 55]. However, the presence of transplanted cells and these effects seem to be transient [44, 54, 55]. The immunosuppressive properties of MSCs were also tested for allograft tolerance in a model of allogeneic heart transplantation, with contrasting results, including increased rejection depending on cell dose and treatment [56, 57]. These opposite conclusions are confusing, and a definitive answer in such a complex field requires further experiments. 

The immunomodulatory effects of ADSCs have been described *in vitro* and *in vivo* [58–62]. These effects could also be due to secreted factors such as transforming growth factor beta, hepatocyte growth factor, prostoglandin E2, and IDO [62] and in some areas, different from MSC effects. Indeed, ADSCs inhibit immunoglobulin production but also suppress this B-cell function to a much greater extent than MSCs [63]. So the use of ADSCs could not be a simple and direct translation of MSC uses and needs dedicated experiments.

### 4.2. Cell Administration

Cells are transplanted most frequently by intratissue delivery, but massive cell death is frequently observed after the injection. To overcome such limitations, alternative cell-delivery systems should be considered. One solution could be to render the cells more resistant to the accompanied stress of the injection. For example, we have shown that preconditioning with melatonin increases the survival, paracrine activity, and efficiency of MSCs when injected intraparenchymally [64]. This effect is a consequence of higher resistance to oxidative stress and secretion of proangiogenic factors. Another promising approach could be to engineer biological cell sheets to efficiently transplant MSCs by respecting host tissue structure. This approach was successful with use of undifferentiated ADSCs in the heart [65]. After transplantation, the engrafted sheet gradually grew to form a thick stratum that included newly formed vessels, undifferentiated cells, and a few cardiomyocytes. Cardiac wall thinning was reversed in the scar area and cardiac function improved. 

Finally, the possibility of targeting cells to various damaged sites by bloodstream delivery is attractive and seems suitable for these cells because they can be distributed to any tissue with no clonal or extensive proliferation at their final destination site [66]. This route of delivery has been investigated in the heart, and pharmacological agents can modulate its efficiency [67].

## 5. Culture Medium

Although increasing reports, including those of clinical trials, have established that MSCs are good candidates for cell-based therapy, aspects of this therapy should be improved. Regarding new therapeutic targets, central nervous system diseases, autoimmune diseases, and lesions of the skin or cornea, phase I and II trials should be developed. Moreover, GMP conditions, particularly relevant safety controls and closed systems, must be fully implemented, and phase I and II trials should be performed with GMP-produced MSCs. If MSCs are effective with this process, phase III trials can be implemented and cell products finally licensed. Our own experience is consistent with this conclusion, and in the next few years, cell therapy with MSCs or ADSCs will be validated and can help treat a large number of diseases ranging from nonunion fracture, to limb ischemia and heart failure, to autoimmune diseases.

## Figures and Tables

**Table 1:** Features of bone-marrow derived mesenchymal stem cells (MSCs) and adipose-derived stem cells (ADSCs).

Feature	MSCs	ADSCs
Tissue sampling	Adult	Adult
	General anesthesia	Local anesthesia
Cell purification process	No proteolytic digestion	Proteolytic digestion, generate different cell subsets
Phenotype	CD49a, CD73, CD90, CD105 CD271 and negative for CD34	CD49a, CD73, CD90, CD105 (CD271?) and CD34 in early passage
Frequency (CFU-Fs)	0.005	0.05
Potency	Hematopoietic support Classic mesenchymal lineage (adipo-, chondro- and osteogenesis) Immunosuppressive properties Precommitted towards osteogenesis (cultured MSCs)	Hematopoietic support Classic mesenchymal lineage (adipo-, chondro- and osteogenesis) Immunosuppressive properties Precommitted towards adipogenesis Good angiogenic potential

CFU-F: colony-forming unit-fibroblasts.
